# Genome-wide identification and expression analysis of Raf-like kinase gene family in pepper (*Capsicum annuum* L.)

**DOI:** 10.1080/15592324.2022.2064647

**Published:** 2022-04-17

**Authors:** Chae Woo Lim, Sung Chul Lee

**Affiliations:** Department of Life Science (BK21 program), Chung-Ang University, Seoul, South Korea

**Keywords:** Abiotic stress, gene expression, mitogen-activated protein kinase, pepper, raf-like kinase

## Abstract

As highly conserved signaling pathway modules, mitogen-activated protein kinase (MAPK) cascades play vital roles in a diverse range of stress and hormonal responses in plants. Among the established components of MAPK cascades, Raf-like MAPK kinase kinases (MAPKKKs) are associated with abscisic acid (ABA) signaling and osmotic stress responses. However, despite the availability of a pepper reference genome, few of the Raf-like kinases in pepper plants have been functionally characterized. In this study, we isolated 47 putative Raf-like kinase genes from the pepper genome based on in silico analysis, which were classified into two major categories, namely, groups B and C (further sub-grouped into B1–B4 and C1–C7, respectively) and named sequentially as *CaRaf1* to *CaRaf47*. Subcellular localization prediction analysis revealed that most of the group B CaRaf-like kinases are probably nuclear-localized, whereas a majority of group C members targeted into the cytoplasm. Transcriptional regulation of the 47 *CaRaf* genes in response to treatment with ABA, drought, NaCl, and mannitol was quantitatively analyzed by reverse-transcription PCR analysis. This revealed a significant induction of subgroup B3, C2, C3, and C5 members, indicating that these genes may be functionally associated with the response to osmotic stress, mediated via both ABA-dependent and -independent pathways. The findings of this study can accordingly serve as a basis for the identification of CaRaf genes associated with the regulation of ABA signaling and osmotic stress response and thus contribute to enhancing our understanding of the biological functions of CaRaf kinases in the responses of plants to different abiotic stresses.

## Text

Protein phosphorylation is a well-established major type of post-translational modification associated with the modulation of numerous signaling pathways. Phosphorylation is catalyzed by several types of protein kinases, among which the mitogen-activated protein kinase (MAPK) family of serine/threonine protein kinases have been extensively studied. Typically, MAPK cascades consist of at least three tiers of kinases, namely, MAPK kinase kinase (MAPKKK, MAP3K, or MEKK), MAPK kinase (MAPKK, MAP2K, MKK or MEK), and MAPK (MPK) kinases.^1^^, 2^ The protein kinases comprising MAPK cascades have been identified in a range of plant species, including *Arabidopsis* and tomato.^3–7^ Although evolutionarily conserved within a plant species, there tends to be broad interspecific comparability regarding the number of different components in MAPK cascades.^4,8^ For example, 80 MAPKKKs, 10 MAPKKs, and 20 MAPKs have been identified in the *Arabidopsis* genome,^3^ whereas tomato contains 89 MAPKKK, 6 MAPKK, and 16 MAPK genes.^7^ As the largest family of MAPK cascade kinases, Arabidopsis MAPKKKs are divided into three subfamilies, namely, MEKK (21 genes), Raf (48 genes), and ZIK (11 genes),^1,9^ among which, the predominant Raf kinases can be broadly classified into two major groups, B and C, which are in turn divided into subgroups B1 to B4 and C1 to C7, respectively.^1^

The MAPK cascades involved in signaling pathways tend to be highly conserved modules that in plants, play vital roles in a broad array of stress and hormonal responses,^8,10,11^ and in particular, several MAPKKKs have been proposed to be associated with abscisic acid (ABA) signaling and drought stress responses. For example, *Arabidopsis* ABA-INSENSITIVE PROTEIN KINASE has been demonstrated to positively regulate ABA responses implicated in primary root growth and stomatal closure.^12^ AtMAPKKK18 appears to play a similarly positive role in drought resistance.^13^ Recently, we isolated the MEKK family kinase CaAIMK1 (*Capsicum annuum* ABA Induce MAP Kinase 1/CaMEKK24) from pepper and demonstrated that silencing of the *CaAIMK1* gene reduced the ABA-dependent drought resistance of pepper plants.^14^ Among the Raf family MAPKKKs, group B Raf kinases have been shown to positively regulate SNF1-related protein kinase 2s (SnRK2s) involved in the regulation of ABA signaling and osmotic stress responses.^15–19^ In addition to ABA-activated SnRK2s, it has also been established that ABA-unresponsive subclass I SnRK2s are similarly activated by three B4 Raf-like kinases, Raf18, Raf20, and Raf24^16^. In contrast to group B members, the group C Raf-like kinases Raf36 and Raf22 have been identified as direct targets of SnRK2 and reported to play a negative role in ABA signaling.^20^

Pepper is a globally cultivated vegetable crop that is used extensively as a spice, medicine, vegetable, and ornament plant.^21,22^ With ongoing changes in world climate and increases in the frequency and severity of extreme weather events, pepper plants may become more susceptible to adverse environmental conditions, such as drought and high salinity, during their lifetimes. In this regard, the release of a *Capsicum* reference genome has accelerated the identification and functional analysis of a range of stress-related genes from pepper.^21–23^ Nonetheless, despite significant progress in the characterization of a number of these genes, there is still comparatively little information available with respect to pepper Raf-like kinase genes and their functional roles in plant responses to abiotic stresses.

**Figure 1.**
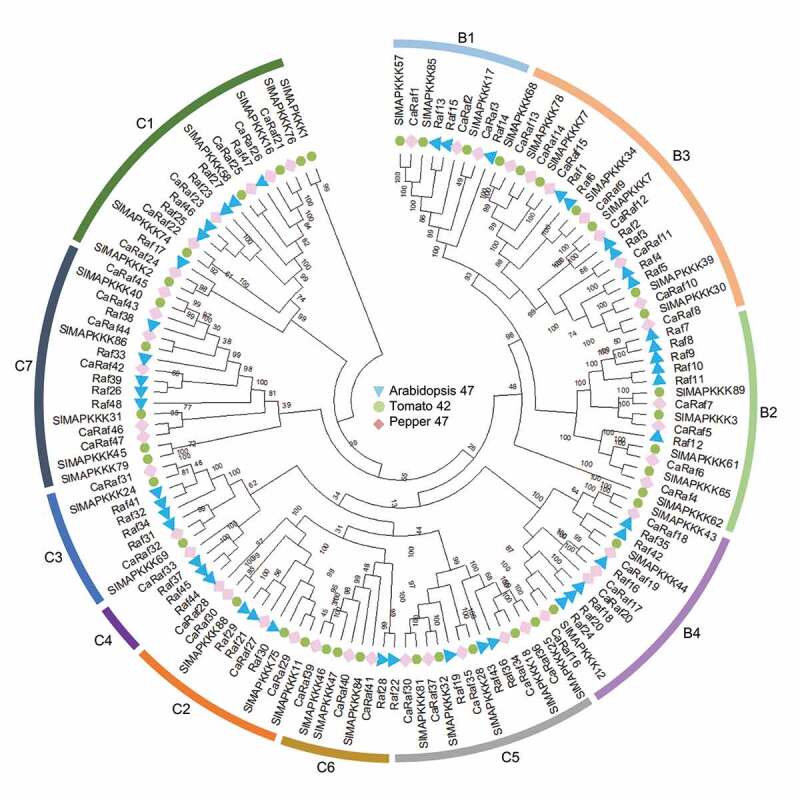
Phylogenetic tree analysis of putative Raf-like kinase gene family in pepper. Amino acid sequences were deduced from the full-length coding sequences of Raf-like kinase genes in pepper (pink diamonds), *Arabidopsis* (sky blue triangles), and tomato (green circles), and were used for comparison. Multiple-sequence alignment and phylogenetic tree analyses were conducted using MEGA X.^24^ The bootstrap consensus tree inferred from 1000 replicates is taken to represent the evolutionary history of the taxa analyzed.^25^ Branches corresponding to partitions reproduced in less than 50% bootstrap replicates are collapsed. Evolutionary distances were computed using the Poisson correction method and values are shown as the number of amino acid substitutions per site. In total 137 amino acid sequences were analyzed, and for each sequence pair, all ambiguous positions were removed (pairwise deletion option).

In this study, we sought to identify pepper Raf-like kinase genes that are associated with ABA signaling and responses to osmotic stress. Initially, BLASTP searches were performed to detect putative Raf-like kinases in the pepper genome. Based on the evolutionary conservation of MAPK cascade components in plants, as queries, we used the full-length amino acid sequences of 47 and 42 Raf kinases from the model plant *Arabidopsis* and tomato respectively, the latter of which is a member of the same Solanaceae family as pepper. As pepper Raf (CaRaf) kinase candidates, we selected 47 homologous sequences (Table 1 and Figure 1). With the exception of CA00g44070, CA00g80950, CA00g68810, and CA00g71750, the chromosomal locations of which we were unable to ascertain, we established that the selected pepper Raf genes are distributed on all pepper chromosomes. Notably, we found the eight genes CA02g10400, CA02g12990, CA02g14110, CA02g16240, CA02g20590, CA02g22960, CA02g23940, and CA02g30080 to be clustered with high density near one of the terminal regions of chromosome 2 (from 129 to 169 Mb).

**Table 1.** List of CaRaf-like kinases in the pepper genome and related information

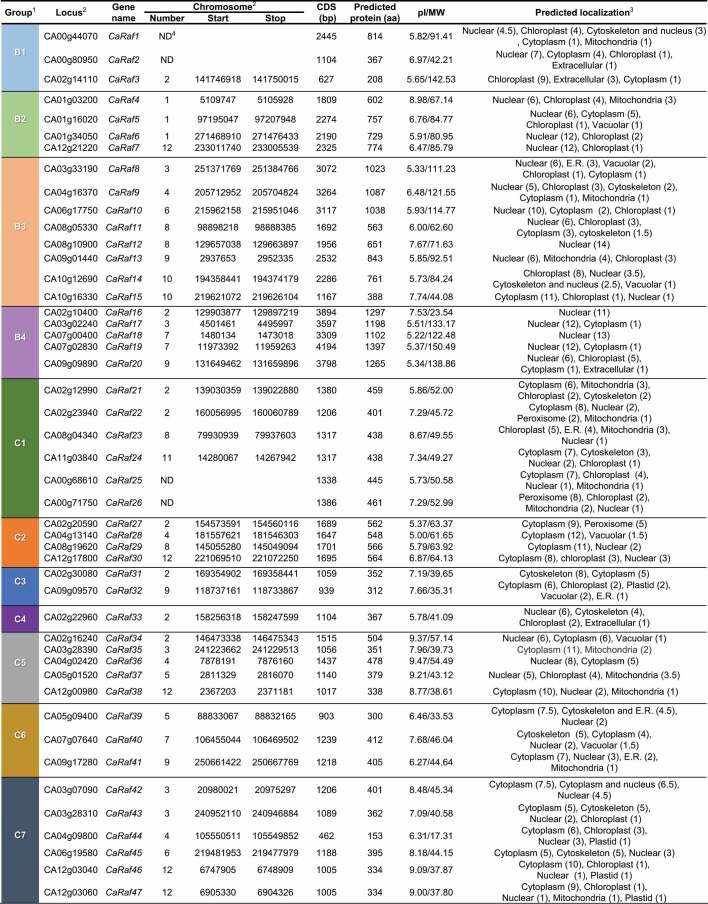

1. Genes are sorted as shown by Ichimura et al^10^.

2. Gene locus and location are from *Capsicum annuum* cv ‘CM334’ genome (release 1.55).

3. Subcellular localization of protein is predicted using a web tool WOLF PSORT (http://www.genscript.com/wolf-psort.html). The number in parenthesis is k value indicating the counts of the nearest neighbors (k used for K-Nearest Neighbors algorithm is 14).

4. ND, not determined due to unsequenced portion of gene.

To analyze evolutionary relationships among the pepper Raf-like kinases, we used MEGA X software to perform phylogenetic tree analysis of the 47 selected kinases, along with amino acid sequences of the aforementioned *Arabidopsis* and tomato Raf family kinases, based on the neighbor-joining method.^24^ The CaRaf proteins were classified into groups B and C and in turn assigned to subgroups B1–B4 and C1–C7, respectively, as established in previous studies.^1,4^ Among these subgroups, C4, comprising a single gene, is the smallest, whereas subgroups B3 (8 genes) and C7 (6 genes) are relatively larger. On the basis of these data, we named the isolated genes sequentially from *CaRaf1* to *CaRaf47*, as shown in Table 1.

**Figure 2.**
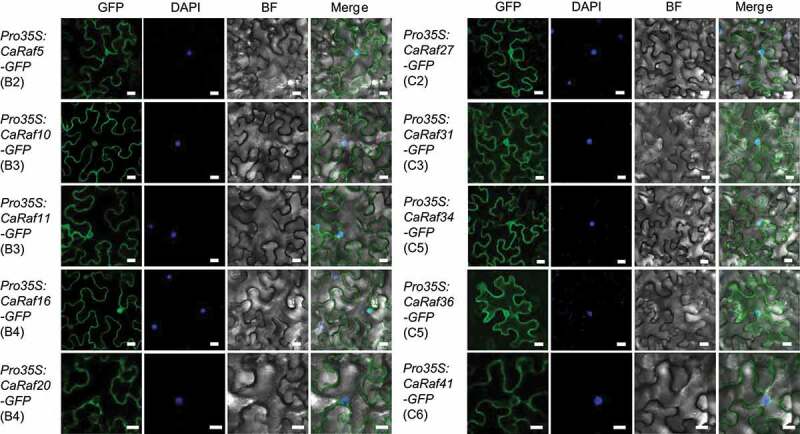
Subcellular localization of CaRaf kinases. GFP-tagged CaRaf kinase proteins were transiently expressed in the leaves of *Nicotiana benthamiana* via *Agrobacterium*-mediated infiltration. Confocal images were taken 2 days after agroinfiltration. White bar = 20 µm.

Given that the subcellular localization of proteins is typically closely associated with function and protein–protein interaction networks, we used WoLF PSORT^26^ to undertake an in silico prediction of subcellular localization. While the majority of B group members are probably predicted to be nuclear-localized, most of the C group members appear to be cytoplasmic proteins. To confirm this, we selected five *CaRaf* kinases from each group and fused them with green fluorescent protein to analyze subcellular localization in the leaves of *Nicotiana benthamiana* via *Agrobacterium*-mediated infiltration. Contrary to our predictions, most proteins, including CaRaf5 (B2), CaRaf10 (B3), CaRaf11 (B3), CaRaf16 (B4), CaRaf27 (C2), CaRaf31 (C3), CaRaf34 (C5), and CaRaf36 (C5), were localized to both nucleus and cytoplasm, whereas CaRaf20 (B4) and CaRaf41 (C6) were predominantly localized to the cytoplasm (Figure 2).

**Figure 3.**
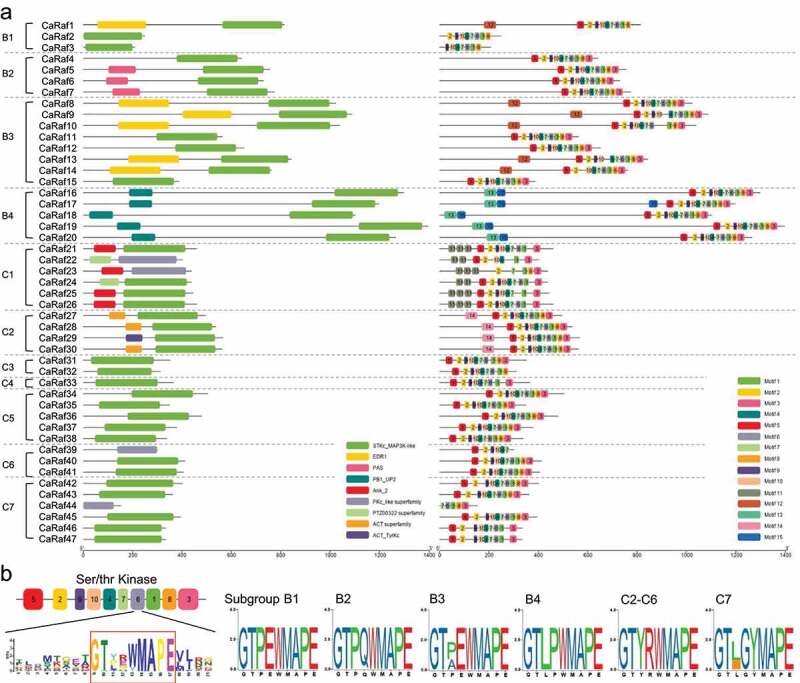
Distribution of conserved domains and motifs in CaRaf-like kinase proteins. (a) A schematic representation of the domain and motif composition of CaRaf-like kinases. Protein domain analysis was based on reference to the NCBI-CDD database (left) and motif analysis was conducted using MEME Suite 5.4.1 (https://meme-suite.org/meme)(right). Graphics were generated using TBtools.^35^ The nine identified conserved domains and ten motif types within the MAP3K-like domains are represented by different colors. Protein lengths are indicated by solid lines and the scale bar is graduated in base pairs (bps). (b) Conserved signature motifs in the kinase domain of Raf-like kinase subgroups. The schematic representation of Motif 6 was generated using the webtool MEME and the sequence logos of the conserved signature motif in each Raf subgroup were generated using TBtools. The height of each letter indicates the probability of an amino acid residue occurring at the respective position.

We subsequently sought to identify domains in the 47 pepper Raf-like protein sequences, based on reference to the NCBI Conserved Domain Database (CDD).^27^ In line with expectations, all proteins were found to contain a Ser/Thr kinase-type catalytic domain (Figure 2a, left). In addition, we identified several domains exclusive to particular subgroups, as follows: a Per-Arnt-Sim (PAS) domain in subgroup B2 kinases, an enhanced disease resistance 1 (EDR1) domain in subgroup B3, and uncharacterized PB1_UP2 domain in subgroup B4, an ankyrin (ANK) repeat domain in subgroup C1, and aspartokinase, chorismate mutase, and TyrA (ACT) domains in subgroup C2. *Arabidopsis* B2 subgroup Raf kinases are similarly characterized by an N-terminal PAS domain, which has been demonstrated to be associated with signal transduction, such as that of light and redox status, in a wide range of organisms due to its sensing properties.^1,28,29^ The EDR1 domain has been defined as a putative non-kinase regulatory region and in *Arabidopsis*, EDR1-containing kinases play negative roles in disease resistance, stress responses, cell death, and ethylene-induced senescence.^30,31^ As one of the most commonly identified conserved domains, ANK is implicated in protein–protein interactions, and ANK-containing kinases have also been reported to be involved in the responses to biotic and abiotic stresses.^32,33^ These findings are thus suggestive of the potential involvement of B2, B3, and C2 subgroup CaRaf kinases in stress responses. We also conducted motif-based sequence analysis to identify the conserved motifs in CaRaf protein domains using the MEME suite tool^34^ with the following parameters: any number of repetitions; a maximum number of 15 motifs; and an optimum width of each motif of between 6 and 50 residues. Motifs 13 and 15, motif 11, and motif 14 were found to be specific to subgroup B4, C1, and C2, respectively (Figure 3a, right), whereas in contrast, motifs 1 to 10 were found within the serine/threonine kinase domain of most CaRaf proteins, For subgroup C1 members, the number of motifs varied from 5 to 9. Among the MAPKKK, Raf subfamily proteins have the conserved signature motif of GTxx(W/Y)MAPE within serine/threonine kinase domain,^9^ and consistent with other plant Raf kinases, CaRaf proteins are also characterized by a highly conserved GTxx(W/Y)MAPE^9^ sequence in motif 6 (Figure 3b). Notably, we found that in the case of subgroup B proteins, the composition of amino acid residues in the signature motif was subgroup specific, The same was also broadly true of C group kinases, with the exception of the C7 subgroup. Furthermore, compared with other subgroups, the subgroup C1 members CaRaf21 to CaRaf26 were observed to contain several amino acid mutations, and although these six proteins have a kinase domain, we do not exclude the possibility that they may not function as Raf kinases.

**Figure 4.**
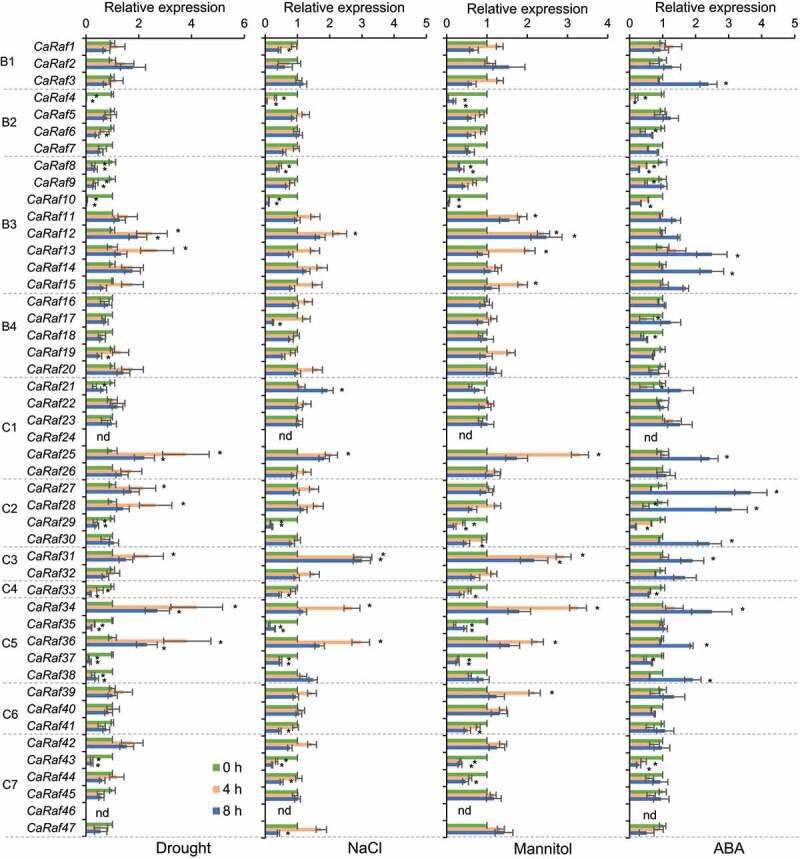
Expression analysis of CaRaf-like kinase genes in the leaves of pepper plants exposed to drought, 250 mM NaCl, 600 mM mannitol, or 100 μM abscisic acid (ABA). The expression of each gene was normalized with that of *CaPP2A* used as an internal control gene, and the value at 0 h was set to 1.0. Data are represented as the means ± standard deviation of three independent experiments. Asterisks indicate significant differences compared with the value at 0 h for each gene (Student’s *t*-test; *P < .05).

Given the established functional assignments for heterologous *Raf* genes in *Arabidopsis*, we speculated as to whether *CaRaf* genes are involved in osmotic stress responses and ABA signaling. To assess this possibility, we performed quantitative reverse transcriptase-polymerase chain reaction (qRT-PCR) analyses to investigate the expression patterns of the 47 selected pepper Raf kinase genes in response to exposure to drought, ABA, NaCl, and mannitol. The germinated seedlings of pepper (*Capsicum annuum* L. ‘Nockwang’) were cultivated in a growth chamber at 25 ± 1°C under white fluorescent light on a 16 h/ 8 h light/dark cycle. Four-week-old pepper plants bearing four fully expanded leaves were used for abiotic stress and chemical treatments. Drought stress was treated by removing plant roots from the soil and then drying them in a growth chamber with 40% relative humidity. For NaCl, mannitol, and ABA treatments, plants were irrigated with 250 mM NaCl and 600 mM Mannitol solution, respectively, and plant leaves were sprayed with 100 μM ABA. Total RNAs were isolated from the first and second leaves of pepper plants harvested after treatment for 4 and 8 h, from which cDNA was synthesized using a Transcript First Strand cDNA Synthesis kit (Roche, Indianapolis, IN, USA), as described previously.^36–38^ qRT-PCR analysis revealed that of the 47 genes, neither *CaRaf24* nor *CaRaf46* was amplified from either of the two timepoint samples, thereby indicating that these could be pseudogenes (Figure 4). Drought stress was observed to influence the expression levels of *CaRaf* genes, particularly those in subgroups B3, C2, C3, and C5, with *CaRaf12* (B3), *CaRaf13* (B3), *CaRaf27* (C2), *CaRaf28* (C2), *CaRaf31* (C3), *CaRaf34* (C5), and *CaRaf36* (C5) being significantly induced, whereas the expression of *CaRaf4* (B2), *CaRaf8* (B3), *CaRaf9* (B3), *CaRaf29* (C2), *CaRaf35* (C5), *CaRaf37* (C5), and *CaRaf38* (C5) was reduced in response to drought stress. With the exception *CaRaf9* (B3), *CaRaf27* (C2), *CaRaf28* (C2), and *CaRaf38* (C5), these same genes showed similar patterns of expression in response to NaCl and mannitol treatments. It has previously been reported that in a number of plant species, endogenous ABA accumulates in response to drought, NaCl, and mannitol treatments,^36,39,40^ and consistently, we found the expression of 11 of the assessed genes to be co-responsive to ABA, drought, NaCl, and mannitol, among which, that of *CaRaf25* (C1), *CaRaf31* (C3) *CaRaf34* (C5), and *CaRaf36* (C5) increased in response to all treatments, whereas there were reductions in the levels of *CaRaf4* (B2), *CaRaf8* (B3), *CaRaf10* (B2), *CaRaf29* (C2), *CaRaf33* (C4), *CaRaf37* (C5), and *CaRaf43* (C7). Collectively, these observations would thus tend to indicate that the expression of these Raf genes is altered in an ABA-dependent manner. In contrast, whereas *CaRaf12* (B3) and *CaRaf35* (C5) were both highly induced by drought, NaCl, and mannitol, they appeared to be less responsive to ABA, thereby indicating that these two *CaRaf* genes may be induced independently of ABA. On the basis of these observations, we can thus speculate that subgroup B3, C2, C3, and C5 CaRaf kinases are functionally associated with osmotic stress via either ABA-dependent or -independent pathways. In this regard, it has previously been established that among *Arabidopsis* Raf kinases, subgroup B2 (RAR7, RAF10, RAF11, and RAF12) and subgroup B3 (RAF3/M3Kδ1, RAF4/M3Kδ7, RAF5/M3Kδ6/SIS8, and RAF6) kinases can phosphorylate and activate the subclass III SnRK2.2, SnRK2.3, and SnRK2.6, core kinases of ABA signaling pathways.^15,16,18,19,41^ Furthermore, it has been demonstrated that mutants with multiple-gene knock-out of osmotic stress-activated subgroup B2 and B3 Raf kinases exhibit ABA hyposensitivity.^19^ In contrast, subgroup B4 members Raf18, Raf20, Raf24, and Raf40 are phosphorylated during the early response to osmotic stress and regulate ABA-independent signaling via the phosphorylation of ABA-unresponsive subclass I SnRKs,^17,42,43^ and triple-knockout mutants of *Raf18, Raf20*, and *Raf24* are characterized by growth retardation in response to osmotic stress.^17^ Additionally, it has been reported that subgroup C5 member Raf43 is involved in ABA responses during seed germination and seedling root growth,^44^ and another C5 kinase Raf22 and a C6 kinase Raf36 play a negative role in the modulation of ABA signaling.^20,45^ In particular, Raf22 and Raf36 are identified as direct targets of subclass III SnRK2s and in response to ABA, SnRK2-mediated Raf36 phosphorylation promotes degradation of Raf36 protein.^20^ In phylogenetic tree analysis, subgroup B2 kinase RAR7, RAF10, and RAF11 are clustered with CaRaf5 and CaRaf7 (Figure 1); by protein sequence identity/similarity calculation, CaRaf5 and CaRaf7 share higher sequence homology with RAF10 (55.9% identity/ 70.9% similarity) and RAF7 (62% identity/ 76.9% similarity), respectively. Subgroup B3 kinase RAF3, RAF4, RAF5, and RAF6 are close to CaRaf11 (31.7% identity/ 40.8% similarity), CaRaf10 (55.9% identity/ 69.6% similarity), CaRaf8 (59.7% identity/ 74.8% similarity), and CaRaf9 (50.3% identity/ 63.1% similarity), respectively. Subgroup B4 kinase Raf18, Raf20, and Raf24 are clustered with CaRaf16; among them, Raf24 shares 45.3% identity/ 61% similarity with CaRaf16. Raf40 are placed in the same clade with CaRaf40, but they share low seqeunce homology (18.2% identity/ 26.2% similarity). For subgroup C, C5 kinase Raf22 and Raf43 and C6 kinase Raf36 are close to CaRaf40 (77% identity/ 86.9% similarity), CaRaf36 (47.2% identity/ 64% similarity), and CaRaf34 (58% identity/ 72.4% similarity), respectively. Given the functional involvement of the *Arabidopsis* Rafs in osmotic stress and ABA signaling, we expected that pepper homologous genes are responsive to stress treatments. However, the pepper genes from subgroups B2, B3, and B4 showed either a significant reduction in expression or little or no change in response to stress treatments. In contrast to subgroup B kinases, C5 kinase *CaRaf34* and *CaRaf36*, except for C6 *CaRaf40*, showed highly significant induction in response to all treatment. This discrepancy between *Raf* gene expression levels and their functions in stress signaling raised the following possibilities: (1) these *Raf* genes may require post-translational modification for activation during plant stress responses and (2) transcriptional alterations of *Raf* genes may be associated with feedback regulation.

In conclusion, in this study, we isolated 47 Raf-like genes from the pepper genome, which could be divided into two major groups, B and C. Furthermore, we established that subgroup B2, B3, C3, and C5 *CaRaf*genes could be associated with responses to osmotic stress and ABA signaling. Functional studies of these genes are currently ongoing, which will provide evidence to indicate which *CaRaf*-like genes contribute to the regulation of osmotic stress resistance, mediated via either ABA-dependent or -independent pathways. Given the post-translational modification of Raf kinases, further studies will not exclude the possibility that the stress-unresponsive pepper Raf genes may also be involved in stress and ABA signaling. Our future research will also focus on the identification of CaRaf-interacting partners, including ABA-responsive and unresponsive SnRK2s, and their up/downstream associations, which will contribute to enhancing our understanding of the activation of ABA-dependent/-independent MAPK cascades in response to osmotic stress.
